# (-)-Epigallocatechin-3-Gallate (EGCG) Modulates the Composition of the Gut Microbiota to Protect Against Radiation-Induced Intestinal Injury in Mice

**DOI:** 10.3389/fonc.2022.848107

**Published:** 2022-04-11

**Authors:** Shang Cai, Li-Wei Xie, Jia-Yu Xu, Hao Zhou, Chao Yang, Lin-Feng Tang, Ye Tian, Ming Li

**Affiliations:** ^1^ Department of Radiotherapy and Oncology, The Second Affiliated Hospital of Soochow University, Suzhou, China; ^2^ Institute of Radiotherapy and Oncology, Soochow University, Suzhou, China; ^3^ State Key Laboratory of Radiation Medicine and Protection, School of Radiation Medicine and Protection, Medical College of Soochow University, Collaborative Innovation Center of Radiation Medicine of Jiangsu Higher Education Institutions, Soochow University, Suzhou, China; ^4^ Department of Nucleus Radiation-related Injury Treatment, Chinese People's Liberation Army Rocket Force Characteristic Medical Center, Beijing, China

**Keywords:** (-)-epigallocatechin-3-gallate, microbiota, radiation-induced intestinal injuries, beneficial bacteria, dysbiosis

## Abstract

The high radiosensitivity of the intestinal epithelium limits the outcomes of radiotherapy against abdominal malignancies, which results in poor prognosis. Currently, no effective prophylactic or therapeutic strategy is available to mitigate radiation toxicity in the intestine. Our previous study revealed that the green tea polyphenol (-)-epigallocatechin-3-gallate (EGCG) attenuates radiation-induced intestinal injury (RIII). The aim of the present study was to determine the effect of EGCG on the intestinal flora of irradiated mice. EGCG administration reduced radiation-induced intestinal mucosal injury, and significantly increased the number of Lgr5^+^ intestinal stem cells (ISCs) and Ki67^+^ crypt cells. In addition, EGCG reversed radiation-induced gut dysbiosis, restored the Firmicutes/Bacteroidetes ratio, and increased the abundance of beneficial bacteria. Our findings provide novel insight into EGCG-mediated remission of RIII, revealing that EGCG could be a potential modulator of gut microbiota to prevent and treat RIII.

## Introduction

Radiotherapy is the main treatment modality against solid tumors using ionizing radiation to kill cancer cells. It is used to treat at least 50% of cancer patients and plays a crucial role in 25% of the cured cases. Acute radiation enteropathy (ARE) occurs within 3 months after radiation in 60-80% of patients who receive pelvic radiotherapy (1). Typical symptoms of ARE include diarrhea, nausea, vomiting, and belly cramps. These symptoms may cause dehydration, electrolyte imbalance, and malnutrition, adversely affecting patient condition at the time of the treatment (2, 3). Unexpected irradiation exposure can result in severe life-threatening intestinal injuries even in healthy individuals (4). Acute illness caused by high dose ionizing radiation is called acute radiation syndrome (ARS) (5), which can be stratified into hematopoietic, gastrointestinal and neurovascular syndrome depending on the dose. So far, effective countermeasures have only been developed against hematopoietic ARS (6); thus, there is an urgent need to develop novel strategies for the prevention or treatment of radiation-induced intestinal injury (RIII).

The polyphenol (-)-epigallocatechin-3-gallate (EGCG) accounts for 50-80% of the total catechin content in green tea. It exhibits potent neuroprotective, hypoglycemic, antioxidant, antibacterial, antiviral, anti-tumor (7, 8) and radioprotective effects in various tissues (9). Additionally, EGCG protects phospholipid molecules against the oxidative damage induced by ultraviolet radiation and blue light irradiation (10–12). Novel nanofiber formulations containing EGCG have the ability to scavenge the toxic reactive oxygen species (ROS) generated by exposure to either H_2_O_2_ or ultraviolet radiation and significantly accelerates the wound-healing process (13). Importantly, our previous study demonstrated that EGCG prevents RIII by scavenging ROS and inhibiting cell death through the Nrf2 signaling pathway (14). Nevertheless, the mechanisms underlying the radioprotective role of EGCG remain to be elucidated.

Our study as well as other studies reported dysbiosis in the gut microbiota following abdominal or total body irradiation, characterized by a decrease in the diversity of gut microbiota with a less relative abundance of beneficial bacteria, and an increase in the relative abundance of opportunistic pathogens (15, 16). Radioprotection based on gut microbiota, such as probiotics, fecal microbiota transplantation and intervention with active compounds, can regulate gut microbiota balance and alleviate RIII in the irradiated animals and patients subjected to radiotherapy to some extent (15, 17–19). The gut microbiota is a key factor in maintaining the intestinal barrier function, which is crucial for nutrient absorption, energy metabolism and immune regulation (20). A study reported that the intestinal microflora metabolizes macromolecular plant polyphenols into more bioactive metabolites and improves their bioavailability, and these metabolites promote the growth of beneficial bacteria instead of pathogenic bacteria (21). Previous studies showed that EGCG is metabolized by intestinal probiotics, and the metabolites subsequently enter the circulation (22). The metabolite trimethylphloroglucinol for instance is detected in the urine at 5 hours after oral administration of EGCG (23). A recent study showed that phloroglucinol inhibits xanthine oxidase, reduces ROS levels and alleviates acute intestinal inflammation (24). However, it is unclear whether the radioprotective effect of EGCG is related to its metabolites or the intestinal microflora.

The present study revealed that EGCG prevented RIII in a mouse model following total body irradiation (TBI) by preserving the intestinal stem cells (ISCs) and increasing the abundance of probiotic species in the gut. Our findings suggest that the gut microbiota plays a crucial role in mediating the radioprotective effects of EGCG.

## Materials and Methods

### Chemicals

EGCG was purchased from Sigma-Aldrich (St Louis, MO, USA) and the stock solution of 8.25 mg/ml was prepared in normal saline.

### TBI and Treatment Regimen

The animal experiments were performed according to the guidelines of Soochow University. Six-week-old male C57BL/6J mice (SLAC Laboratory Animal Co. Ltd, Shanghai, China) were acclimatized for 7 days before the experiments. The animals were housed in plastic cages (4 or 5 mice/cage) under controlled humidity (44 ± 5%), light (12 hour light/dark cycles) and temperature (22 ± 2°C), with free access to drinking water and food. The mice were randomly divided into three groups as follows (n = 6 for each time point): 1) Control, 2) IR + vehicle and 3) IR + EGCG. The mice in group 1 were sham irradiated. The mice in groups 3 and 2 were treated with an oral administration of 25 mg/kg EGCG or an equivalent volume of saline for 5 consecutive days before 9 Gy TBI, and 30 minutes after TBI. Radiation was administered through a ^60^Co γ source at the dose rate of 2 Gy/min.

### Histological Staining

Jejunum tissues were isolated at the specific time points, flushed with ice cold phosphate-buffered saline (PBS), and fixed overnight with 10% neutral formalin before paraffin embedding. Three micrometer-thick sections were stained with hematoxylin and eosin (H&E) and examined under a light microscope. The villus height and crypt depth were analyzed from the images using the Image J 1.43 software. A minimum of 30 well-oriented, full-length crypt-villus units per mouse were measured, and crypts per circumference were counted in three sections per mouse.

### Immunohistochemistry (IHC)

Tissue sections were dewaxed, heated in citrate buffer for antigen retrieval, and treated with hydrogen peroxide for 15 minutes to quench endogenous peroxides. After blocking with 5% bovine serum albumin for 30 minutes, the sections were incubated overnight with the primary antibodies anti-Lgr5 (1: 200; Abbiotec, San Diego, CA, USA) and anti-Ki67 (1: 400; Cell Signaling Technology, Beverly, MA, USA) at 4°C. The sections were thoroughly washed with PBS and then incubated with the secondary antibody for 30 minutes at 37°C. After another wash, the staining was developed using a DAB kit (Zhongshan Golden Bridge Biotechnology, Beijing, China), and the sections were counterstained with hematoxylin.

### Fecal DNA Extraction and 16S rRNA Gene Sequencing

The fecal samples of each mouse were freshly collected at 3 days after TBI, and then immediately placed into sterile plastic tubes on ice and frozen at -80 °C. Total genomic DNA was extracted using the sodium dodecyl sulfate-based method. The V3-V4 region of the bacterial 16S ribosomal RNA gene was amplified using the following primers: 343F 5′-barcode-TACGGRAGGCAGCAG-3′ and 798R 5′-barcode-AGGGTATCTAATCCT-3′, where the barcode is an eight-base sequence unique to each sample. The PCR products were purified using Agencourt AMPure XP beads (Beckman Coulter Co., Brea, CA, USA) and sequenced on the Illumina Miseq sequencing platform (Illumina Inc., San Diego, CA, USA) by OE Biotech Co. Ltd. (Shanghai, China). The raw data were uploaded to the NCBI Sequence Read Archive (SRA) database under the BioProject accession number PRJNA748836.

### Sequencing Data Analysis

The operational taxonomic units (OTUs) were defined with a threshold of 97% identity by the Uparse software. Analysis was performed separately at each taxonomical level (phylum, class, order, family and genus). Species annotation analysis was conducted using the Mothur method by the SSUrRNA database. Alpha and beta diversity were determined for each library using QIIME software (Version 1.9.1). LEfSe software was used to perform LEfSe analysis. Unweighted unifrac for Principal Coordinate Analysis (PCoA) and Unweighted Pair Group Method with Arithmetic mean (UPGMA) clustering tree were used to assess the variation between the experimental groups (beta diversity). Alpha diversity was calculated for all the samples.

### Statistical Analysis

Results are presented as mean ± SEM. Multiple groups were compared by the analysis of variance (ANOVA) and Tukey’s *t*-test. *P* < 0.05 was considered statistically significant.

## Results

### EGCG Attenuated RIII in Mice by Promoting the Proliferation of Crypt Cells and the Survival of ISCs

**Figure 1:**
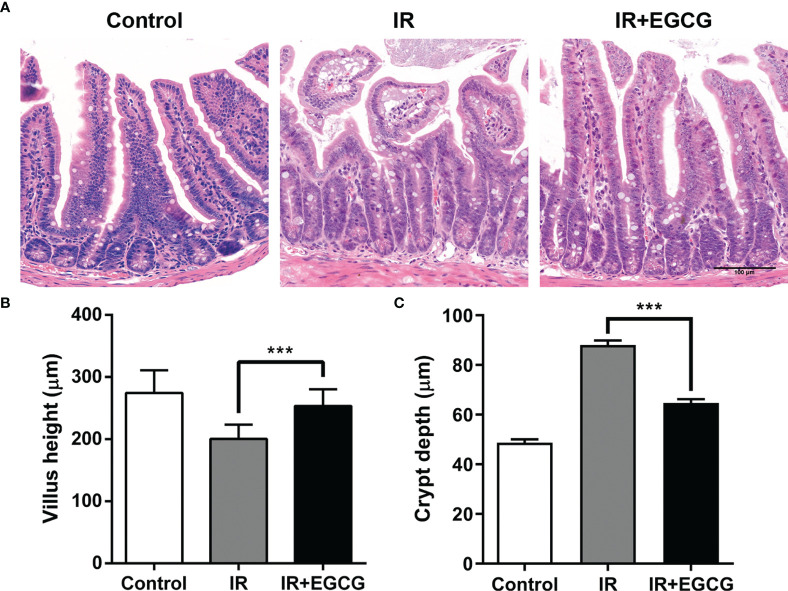
EGCG attenuates radiation-induced intestinal injury in mice. **(A)** Representative H&E images of intestinal sections of the control and irradiated mice at 5 days after 9 Gy TBI (Scale bar = 100 μm). Bar graph showing the villus height **(B)** and crypt depth **(C)** in the indicated groups. All villi and crypts around a circumference were measured per sample. Results are presented as mean ± SEM. ***p < 0.001; n = 6.

**Figure 2:**
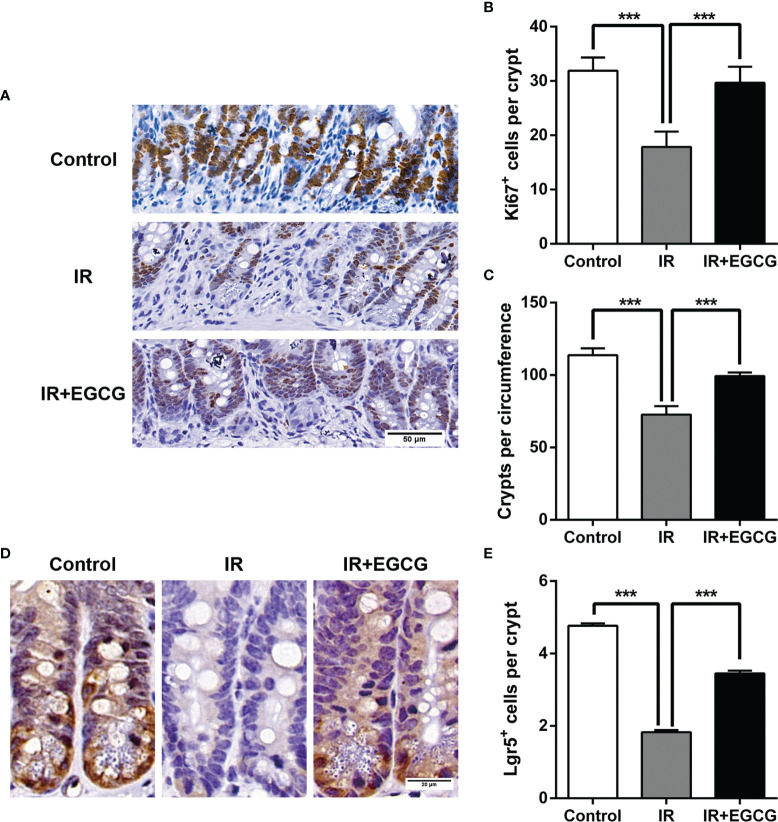
EGCG promotes the proliferation of crypt cells and the survival of ISCs after radiation. **(A)** Representative IHC images of intestinal tissues showing the *in situ* expression of Ki67 in the indicated groups at 5 days after TBI (Scale bar = 50 μm). **(B, C)** Histograms showing the number of Ki67^+^ cells per crypt and surviving crypts per circumference in the different groups. The crypts with more than 10 Ki67^+^ cells were considered as surviving crypts. **(D)** Representative IHC images showing the *in situ* expression of Lgr5 in the indicated groups at 5 days after TBI (Scale bar = 20 μm). **(E)** Histogram showing the number of Lgr5^+^ ISCs per crypt at 5 days post-TBI. Results are presented as mean ± SEM. ***p < 0.001; n = 6.

Mice were subjected to 9 Gy TBI with or without EGCG treatment to assess the potential radioprotective effects of EGCG. The radiation exposure significantly deteriorated the intestinal villi and led to crypt loss compared to the non-irradiated tissues. In contrast, the crypt-villus architecture of the intestinal tracts in the EGCG-treated mice was well-preserved (**Figure 1A**). Furthermore, the villus height and crypt depth were severely affected in the irradiated mice, and EGCG treatment not only dramatically lessen the damage in the unhealthy villi (*p* < 0.001, *t* = 13.16, *F* = 1.38; **Figure 1B**), but also restored the crypt depth (*p* < 0.001, *t* = 9.88, *F* = 1.45; **Figure 1C**). Consistent with the histological findings, the number of actively proliferating Ki67^+^ cells per crypt were significantly higher in the EGCG-treated mice 5 days post-TBI compared to the vehicle control group (29.8 ± 2.9 *vs.* 17.8 ± 2.8; *p* < 0.001, *t* = 42.08, *F* = 1.08; **Figures 2A, B**), and was close to that in the normal controls (31.4 ± 2.4). Furthermore, the mean number of Ki67^+^ crypts per circumference significantly decreased after TBI and was increased in the EGCG-treated group (*p* < 0.001, *t* = 10.16, *F* = 5.60; **Figure 2C**). The actively circulating ISCs are essential for the intestinal regeneration after radiation injury and are characterized by the surface expression of leucine-rich repeat-containing G-protein coupled receptor 5 (Lgr5) (25). TBI markedly reduced the number of Lgr5^+^ ISCs per crypt compared to the controls, which recovered partially in the EGCG-treated mice at 5 days after TBI (*p* < 0.001, *t* = 17.22, *F* = 1.27; **Figures 2D, E**). Taken together, the results showed that EGCG attenuated the radiation-induced structural damage in the small intestines by preserving ISCs and enhancing intestinal epithelial cell regeneration.

### EGCG Reversed TBI-Induced Gut Dysbiosis

**Figure 3:**
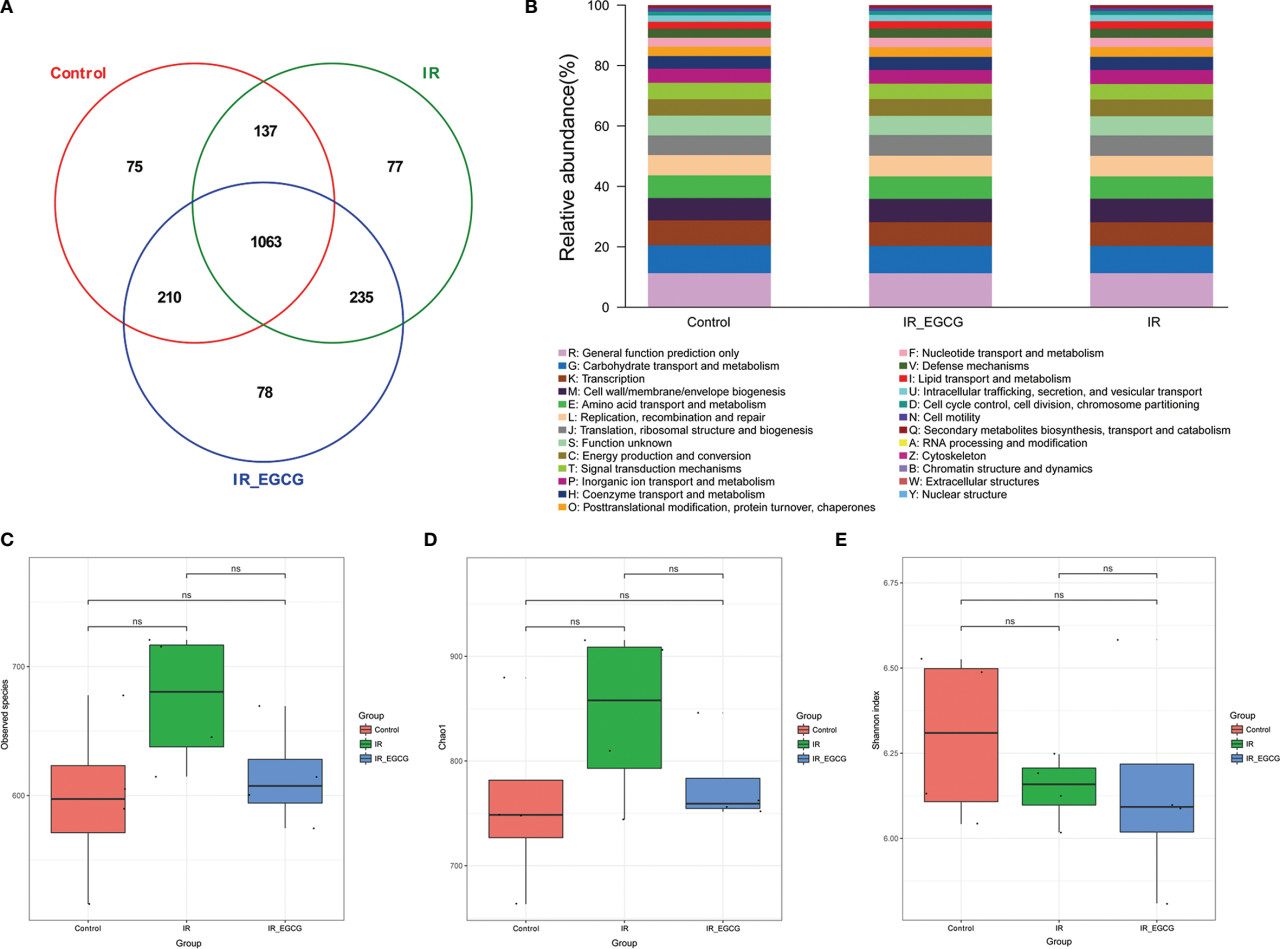
Effects of EGCG treatment on gut bacterial composition. **(A)** Venn diagram showing the common and unique OTUs across all groups. The numbers in the Venn diagram indicate the number of OTUs. **(B)** Relative abundance of functional categories in gut microbiota among the three groups as predicted by Functional Annotation of Prokaryotic Taxa (FAPROTAX). The observed species number **(C)**, Chao1 index **(D)** and the Shannon diversity index **(E)** of the intestinal bacterial flora. Results are presented as mean ± SD. ns, not significant; n = 4.

**Figure 4:**
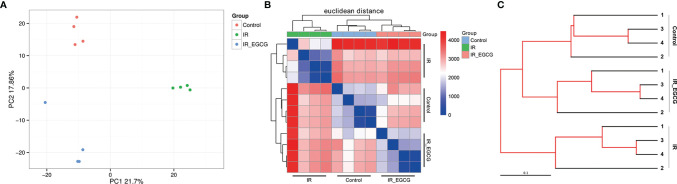
EGCG treatment restores the composition of the intestinal flora in the irradiated mice. **(A)** Differences in gut microbiota composition in vehicle/EGCG-treated mice after the exposure to 9 Gy TBI assessed by principal coordination analysis (PCoA). **(B)** Heatmap calculated from the Euclidean distance of the fecal samples of all groups. **(C)** UPGMA tree of the gut microbiota from vehicle/EGCG-treated mice after the exposure to 9 Gy TBI.

The consumption of tea rich in polyphenols is beneficial for the intestinal microecology and host health (26). The composition of gut microbiota was analyzed through *16S rRNA* gene sequencing to determine the effect of EGCG on the gut microflora of irradiated mice. A total of 1063 bacterial OTUs were shared among the control, IR and IR + EGCG groups, and the latter had the highest number of unique OTUs (**Figure 3A**). However, as predicted by the Functional Annotation of Prokaryotic Taxa, no significant difference in the relative abundance of functional categories was observed in the gut microbiota across the three groups (**Figure 3B**). Furthermore, TBI had no effect on the number of enteric bacterial species (**Figure 3C**) or the alpha diversity, which was confirmed by Chao1 index and Shannon diversity index analyses (**Figures 3D, E**). The repeatability of samples within a group, and the similarities and differences among the groups were assessed in terms of the beta diversity by PCoA and UPGMA analyses. The results of PCoA analysis indicated a clear separation among the three groups, indicating that the composition of gut microbiota was substantially altered after TBI and partially restored by EGCG (**Figure 4A**). The Euclidean distance and UPGMA analysis also revealed distinct gut microbiota of the control and irradiated groups, and the radiation-induced differences were leveled by the EGCG treatment (**Figures 4B, C**). Taken together, the results showed that EGCG restored TBI-altered gut microbiota, which could be the basis of its radioprotective effects.

### EGCG Increased the Abundance of Probiotic Species in the Gut After TBI

**Figure 5:**
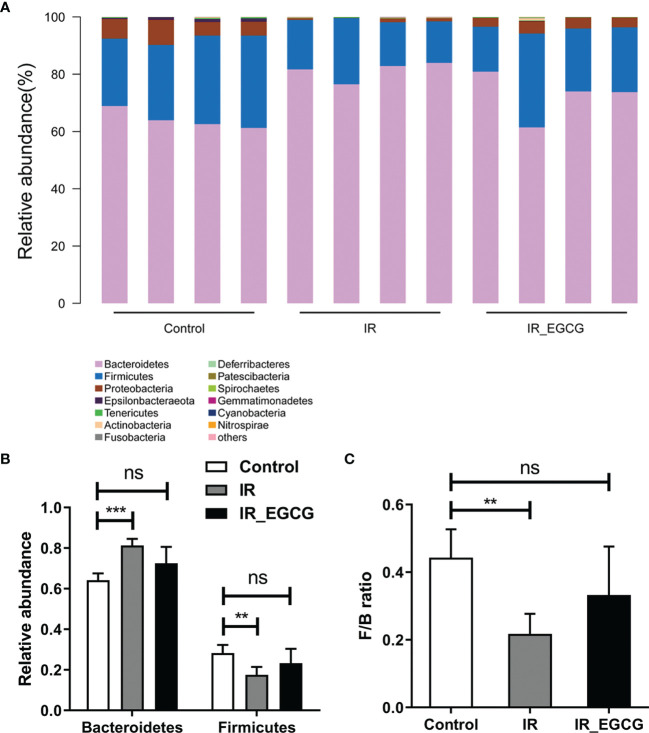
EGCG modulates the composition of gut microbiota at the phylum level. **(A)** Distribution of bacterial phyla across all groups. **(B)** Relative abundance of Firmicutes and Bacteroidetes in fecal samples. **(C)** Firmicutes/Bacteroidetes ratios in the three groups. Results are presented as mean ± SEM. **p < 0.01; ***p < 0.001; ns, not significant; n = 4.

**Figure 6:**
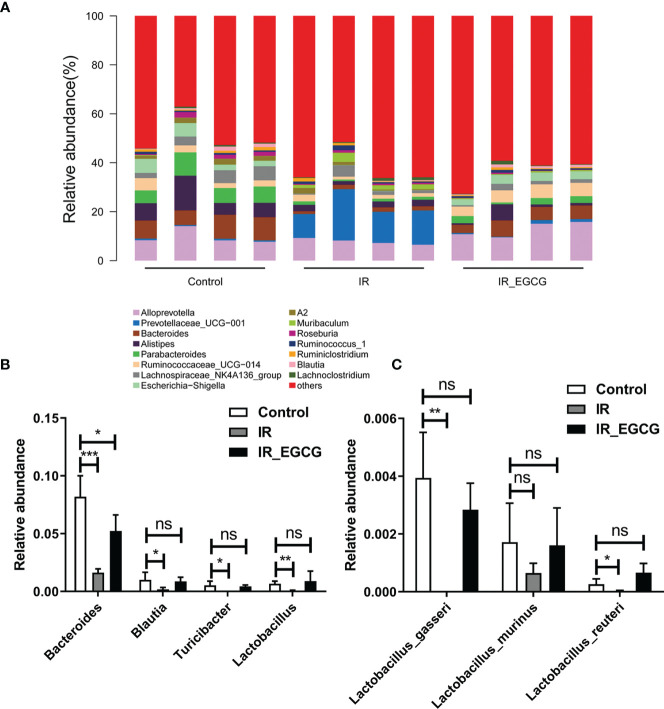
EGCG modulates the gut microbiota composition at the genus level. **(A)** Distribution of bacterial genera across all groups. **(B)** Relative abundance of *Bacteroidales*, *Blautia*, *Turicibacter* and *Lactobacillus* in the fecal samples. **(C)** Relative abundance of *Lactobacillus* after EGCG treatment. Data are represented as the mean ± SEM. *p < 0.05; **p < 0.01; ***p < 0.001; ns, not significant; n = 4.

**Figure 7:**
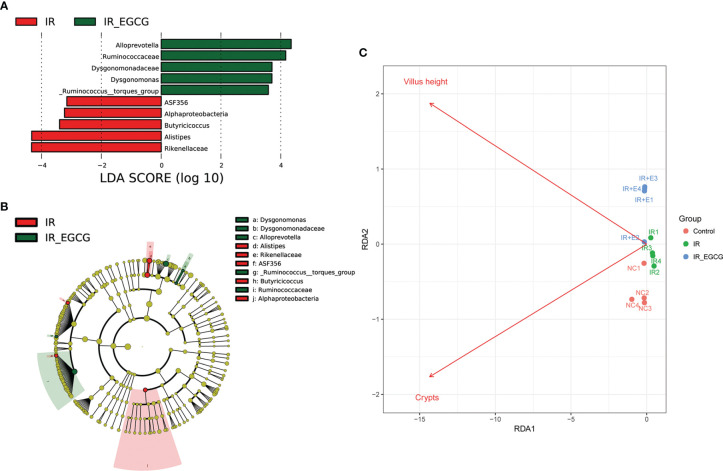
Gut microbiota alterations correlated with TBI-induced intestinal injury. **(A)** Histogram showing the LDA scores indicative of differentially abundant bacteria between the IR (red) and IR + EGCG (green) groups (n = 4). Characteristic taxonomies with LDA scores > 3.5 are shown. **(B)** LEfSe cladogram showing the most differentially abundant taxa between the two groups. The taxa enriched in the IR and IR + EGCG groups are shown in red and green, respectively. The brightness of each dot is proportional to its effect size. **(C)** Redundancy analysis (RDA) of the microbial community structure based on *16S rRNA* gene sequencing data. The length of the arrows corresponds to the influence of the indicated environmental parameter on the microbial communities.

The relative abundance of the predominant taxa in the three groups was analyzed to profile the specific changes in gut microbiota. The relative abundance of the phylum Bacteroidetes was sharply increased in the IR group compared to the controls as shown in **Figures 5A, B** (*p* < 0.001, *t* = 7.26, *F* = 1.02), and was unaffected by EGCG treatment. In addition, TBI significantly decreased the relative abundance of the phylum Firmicutes compared to the control group (*p* < 0.01, *t* = 3.81, *F* = 1.06; **Figures 5A, B**), which was restored to near normal levels by EGCG. The Firmicutes to Bacteroidetes (F/B) ratio is an indicator of the degree of intestinal inflammation and general physiological state (27). TBI decreased the F/B ratio in the gut microbiota (*p* < 0.01, *t* = 4.42, *F* = 2.00; **Figure 5C**), which was normalized by the EGCG treatment. The gut microbiota was further analyzed at the genus and species levels to explore a potential effect of EGCG on the probiotic species. The relative abundance of the general *Bacteroidales* (*p* < 0.001, *t* = 7.13, *F* = 32.30), *Blautia* (*p* < 0.05, *t* = 2.41, *F* = 15.95), *Turicibacter* (*p* < 0.05, *t* = 2.83, *F* = 12.03) and *Lactobacillus* (*p* < 0.01, *t* = 5.01, *F* = 37.55) was significantly decreased in the IR group compared to the controls (**Figures 6A, B**), and was increased in the EGCG-treated animals (**Figures 6A, B**). *Lactobacillus* spp. is highly beneficial in mice and accelerates the regeneration of ISCs after RIII (28). At the species level, EGCG treatment increased the relative abundance of *Lactobacillus gasseri*, *L. murinus* and *L. reuteri* to reach a level similar to that in the control mice (**Figure 6C**). Linear discriminant analysis (LDA) coupled with effect size analysis confirmed that Alloprevotella and Dysgonomonas were the key phyla in the IR+EGCG group, whereas the dominant phyla in the IR group were Rikenellaceae and Alistipes (**Figures 7A, B**). Redundancy analysis (RDA) further showed that villus height and the number of crypts were significantly correlated with the composition of gut microbiota (**Figure 7C**). Overall, EGCG restored the gut F/B ratio and increased the abundance of probiotics in irradiated mice.

## Discussion

Green tea is a popular traditional beverage in East Asia and has numerous health benefits. Regular consumption of green tea may prevent cancer, diabetes, atherosclerosis, obesity, as well as bacterial and viral infections. Both *in vitro* and *in vivo* studies showed that green tea reduces the radiation-induced damage to cells and tissues (14, 29). The present study revealed that the administration of EGCG prior to TBI preserved the crypt-villus architecture of the murine small intestine by promoting the survival of the Lgr5^+^ ISCs and the regeneration of the intestinal epithelium.

Multiple clinical trials demonstrated that the oral administration of EGCG is an effective and safe method to deal with acute radiation esophagitis (30–33). Topical EGCG is an effective treatment for radiation-induced dermatitis and has acceptable toxicity (34). Furthermore, epigallocatechin-3-gallate mouthwash protects mucosa from radiation-induced mucositis in head and neck cancer patients (35). Thus, EGCG is a promising medical countermeasure against RIII and could be considered for clinical use during pelvic radiotherapy and accidental radiation exposure. However, further basic and clinical studies should be performed to confirm and clarify the mechanisms of differential effect of EGCG on cancer and normal tissues during radiation.

EGCG inhibits the proliferation of the intestinal pathogens such as *Clostridium perfringens*, *Clostridium difficile* and *Bacaeroides*, and has a positive effect on probiotics including *Bacillus bifidus* and *Lactobacillus* (36). Furthermore, EGCG affects the composition of the intestinal bacteria in humans as well as animal models and regulates the metabolism (37, 38). Nevertheless, the role of gut microbiota in the radioprotective role of EGCG has not been completely elucidated. Studies showed that the intestinal bacteria deconjugate and degrade EGCG *in vitro* and *in vivo*. For instance, EGCG is almost completely metabolized in the porcine cecum by the resident bacteria within 4-8 hours (39). Furthermore, the radioactivity level in the blood and tissues of rats fed with isotope-labeled EGCG is low after 4 hours and start to increase after 8 hours, reaching peak levels at 24 hours (40). This suggested that EGCG is metabolized by the gut microbiota before being absorbed.

Recent studies showed that the gut microbiota is closely related to the efficacy and side-effects of radiotherapy. Nam et al. (41) found that the abundance of Firmicutes decreases by 10% and that of Fusobacterium increases by 3% after pelvic radiotherapy. Furthermore, Wang et al. (42) reported a decrease in the relative abundance of Bacteroides and a concomitant increase in that of Proteobacteria in patients with radiation enteritis. In a large pilot study of the microbiome in acute and late-radiation enteropathy, a consistent association is observed between low bacterial diversity and late radiation enteropathy, although without statistical significance. Specifically, the increased abundance of *Clostridium* IV, *Roseburia* and *Phascolarctobacterium* is correlated with radiation enteropathy (43). Barker et al. (44) reported that radiotherapy alters the composition of the gut microbiota, disrupts the intestinal barrier and induces apoptosis in the crypt cells. In contrast, Gosiewski et al. (45) showed that therapeutic doses of radiation do not significantly affect *Lactobacillus* populations. Our previous study found that the composition of gut microbiota is correlated with the radiation dose after 3.5 days of radiation. The relative abundances of phylum Proteobacteria, genera *Escherichia-Shigella* and *Eubacterium xylanophilum*_group, and species *Lactobacillus murinus* have a linear correlation with the radiation dose (15). In this study, radiation significantly altered the composition of the murine gut microbiota and decreased the F/B ratio, which was attenuated by EGCG treatment.

EGCG also increased the abundance of probiotic genera such as *Bacteroides*, *Blautia*, *Turicibacter* and *Lactobacillus*. *Blautia* is a genus of anaerobic bacteria that are frequently detected in mammalian intestines and feces (46). *Turicibacter* and *Lactobacillus* are involved in the production of lactic acid *via* fermentation, which promotes the regeneration of intestinal epithelial cells (28, 47). Green tea catechins increase the abundance of Bacteroides and Lactobacillus (37, 48), and the consumption of green tea promotes the growth of other beneficial gut bacteria such as Akkermansia and Bifidobacterium (49). Furthermore, *Lactobacillus acidophilus*, *L. casei* and *Bifidobacterium* spp. reduce symptoms of radiation-induced gut toxicity, such as diarrhea (50). Our previous study found that the administration of probiotics including *Lactobacillus* and *Bifidobacterium* improves the survival of lethally irradiated mice, alleviates RIII and partially restores the diversity of gut microbiota (15). In this study, the increased intestinal abundance of Bacteroides and other beneficial bacteria following EGCG administration indicated that dietary supplementation with EGCG could improve gut microbiota dysbiosis.

Takagaki et al. (51) reported that EGCG is hydrolyzed to EGC and gallic acid in the rat intestine by the resident microflora including *Enterobacter aerogenes*, *Raoultella planticola*, *Klebsiella pneumoniae* subspecies (subsp.) pneumoniae and *Bifidobacterium longum* subsp. Infantis. Furthermore, the degradation of EGCG by intestinal bacteria produced 5-(3’,5’-dihydroxyphenyl)-γ-valerolactone that is subsequently absorbed, and its glucuronid form is the main urinary metabolite (40). Kohri et al. (52) detected the metabolite 4’,4’’-di-O-methyl-EGCG in rats after intravenous administration, and Lambert et al. (53) detected 4’’-O-methyl-EGCG and 4’,4’’-di-O-methyl-EGCG in mouse small intestine, colon, liver and prostate after intragastrical administration. Thus, the metabolic pathways of EGCG are likely dependent on the route of administration. Further microbiota and metabolomics studies are needed to determine the effect of the gut microbiota on EGCG metabolism and the beneficial effects of the circulating metabolites.

In conclusion, EGCG attenuated the damage of the intestinal structure and microbiota induced by radiation and increased the abundance of key probiotics that are likely involved in intestinal regeneration. Therefore, our findings provide novel insights into the health benefits of EGCG and other green tea phenolics.

## Data Availability Statement

The datasets presented in this study can be found in online repositories. The names of the repository/repositories and accession number(s) can be found below: https://www.ncbi.nlm.nih.gov/, PRJNA748836.

## Ethics Statement

The animal study was reviewed and approved by the ethics committee of Soochow University.

## Author Contributions

ML and YT conceived and designed the study. SC and L-WX coordinated and performed most of the experimental work. J-YX and L-FT performed the histological analysis. HZ and CY performed the statistical analyses. ML and SC wrote the manuscript, and YT provided a critical review. All authors approved the final version of the manuscript.

## Funding

This study received funding from the Jiangsu Provincial Key Project in Research and Development of Advanced Clinical Technique (BL2018657), Suzhou Radiotherapy Clinical Medical Center (Szlcyxzx202103), Young Talent Support Project of the 2nd Affiliated Hospital of Soochow University (XKTJ-RC202007), Science Foundation of Jiangsu Health Commission (M2021081) and Research project of medical talent of Suzhou (GSWS2021025). The funder was not involved in the study design, collection, analysis, interpretation of data, the writing of this article or the decision to submit it for publication.

## Conflict of Interest

The authors declare that the research was conducted in the absence of any commercial or financial relationships that could be construed as a potential conflict of interest.

## Publisher’s Note

All claims expressed in this article are solely those of the authors and do not necessarily represent those of their affiliated organizations, or those of the publisher, the editors and the reviewers. Any product that may be evaluated in this article, or claim that may be made by its manufacturer, is not guaranteed or endorsed by the publisher.

## Abbreviations

EGCG, (-)-Epigallocatechin-3-gallate; ISCs, Intestinal stem cells; ARE, Acute radiation enteropathy; ARS, Acute radiation syndrome; RIII, radiation-induced intestinal injury; ROS, Reactive oxygen species; TBI, Total body irradiation; PBS, Phosphate-buffered saline; H&E, Hematoxylin and eosin; SRA, Sequence read archive; OTUs, Operational taxonomic units; PCoA, Principal coordinate analysis; UPGMA, Unweighted pair group method with arithmetic mean; ANOVA, Analysis of variance; Lgr5, Leucine-rich repeat-containing G-protein coupled receptor 5; LDA, Linear discriminant analysis; FAPROTAX, Functional Annotation of Prokaryotic Taxa; RDA, Redundancy analysis; F/B, Firmicutes/bacteroidetes.
